# 

**Published:** 1850-09

**Authors:** 


### CONTENTS:

### PART I.—ORIGINAL COMMUNICATIONS.

Davis' Medical History, Chapter II., continued, - - - - - 181
ART. 1.—Case of Colloid Degeneration of Right Lung, - - - - - 197
ART. 2.—Chloroform in Surgical Operations. By John Halderman, M. D., - - 201
ART. 3.—Sheldon on Congestive Fever, - - - - - - 206
ART. 5.—Collodion in Mammary Inflammations. By John Evans, M. D., - - 213
ART. 6.—Chamber's case of Neuralgia Uteri, - - - - - - 216
ART. 7.—Case of Colloboma Iridis. By A. Garwood, M. D., - - - - 217
ART. 8.—Iod. Potassium in Hydrocephalus. By Valentine Mott Satterlee, M. D., - - 218
ART. 9.—MacArthur on Habitual Costiveness, - - - - - 218
ART. 10.—Case of Tubal Fœtation. By C. C. Warner, M. D., - - - - 220
ART. 11.—Wisconsin State Medical Society, - - - - - - 222
ART. 12.—Western Medical Society of Wisconsin, - - - - - 224
ART. 13.—Medical Society of the Upper Illinois, - - - - - 225

### PART II.—REVIEWS AND NOTICES OF NEW WORKS.

ART. 1.—Lectures on Venereal and other Diseases arising from Sexual Intercousse. By M. Ricord. Translated by Victor de Meiric,- - - - - 227
ART. 2.—Bell on Baths, the Watery Regimen, Hydropathy, Pulmonary Inhalation, &c., - 228
ART. 3.—Maclise' Surgical Anatomy, - - - - - - 229
ART. 4.—Proceedings of various Medical Societies, - - - - - 229
ART. 5.—Report of a suit for Libel, - - - - - - - 233

### PART III.—SELECTIONS.

ART. 1.—Twins Singularly Connected. By Napoleon B. Anderson, M. D.. - - 235
ART. 2.—Treatment of Intermittent Fevers by a single dose of Quinine, - - - 236
ART. 3—Conclusions respecting Valvular Diseases of the Heart. By Dr. A. W. Barclay, - 237
ART. 4.—Nitrate of Silver in Epidemic Dysentery, by Lewis Slusser, M. D., - - 238
ART. 5.—Ministers and Physicians, - - - - - - - 241
ART. 6.—Nelligan's Treatment of Eruptive Diseases, - - - - - 243
ART. 7.—Cure of Scabies by Inunction with Lard, - - - - - 244
ART. 8.—New Method of Treating Hooping Cough. By Dr. Eben. Watson, - - 244
ART. 9.—Allison on the Use of Tannin in Various Diseases, - - - - 247
ART. 10.—Cornell on Epilepsy, - - - - - - - 251
ART. 11.—Sciatica cured by Chloroform, - - - - - - 252

### PART IV.—EDITORIAL.

ART. 1.—Annual Circulars of Medical Colleges, - - - - - 255
ART. 2.—Free Medical Schools, - - - - - - - 258
ART. 3.—New Medical Periodicals, - - - - - - - 260
ART. 4.—Illinois Ge eral Hospital of the Lakes, - - - - - 263
ART. 5.—Cholera in Chicago, - - - - - - - 264
ART. 6.—Miscellaneous Medical Intelligence,- - - - - - 264
ART. 7.—Obituary, - - - - - - - - - 266

